# Significance of extended sports cardiology screening of elite handball referees

**DOI:** 10.1371/journal.pone.0249923

**Published:** 2021-04-09

**Authors:** Orsolya Kiss, Mate Babity, Attila Kovacs, Judit Skopal, Hajnalka Vago, Balint Karoly Lakatos, Csaba Bognar, Reka Rakoczi, Mark Zamodics, Lorinc Frivaldszky, Anna Menyhart-Hetenyi, Zsofia Dohy, Csilla Czimbalmos, Liliana Szabo, Bela Merkely

**Affiliations:** Heart and Vascular Center, Semmelweis University, Budapest, Hungary; Rutgers New Jersey Medical School, UNITED STATES

## Abstract

The significance of cardiology screening of referees is not well established. Cardiovascular risk factors and diseases were examined in asymptomatic Hungarian elite handball referees undergoing extended screening: personal/family history, physical examination, 12-lead ECG, laboratory tests, body-composition analysis, echocardiography, and cardiopulmonary exercise testing. Holter-ECG (n = 8), blood pressure monitorization (n = 10), cardiac magnetic resonance imaging (CMR; n = 27) and computer tomography (CCT; n = 4) were also carried out if needed. We examined 100 referees (age: 29.6±7.9years, male: 64, training: 4.3±2.0 hours/week), cardiovascular risk factors were: positive medical history: 24%, overweight: 10%, obesity: 3%, dyslipidaemia: 41%. Elevated resting blood pressure was measured in 38%. Stress-ECG was positive due to ECG-changes in 16%, due to elevated exercise blood pressure in 8%. Echocardiography and/or CMR identified abnormalities in 19%. A significant number of premature ventricular contractions was found on the Holter-ECG in two cases. The CCT showed myocardial bridge or coronary plaques in one-one case. We recommended lifestyle changes in 58%, new/modified antihypertensive or lipid-lowering therapy in 5%, iron-supplementation in 22%. By our results, a high percentage of elite Hungarian handball referees had cardiovascular risk factors or diseases, which, combined with physical and psychological stress, could increase the possibility of cardiovascular events. Our study draws attention to the importance of cardiac screening in elite handball referees.

## Introduction

The routine screening of athletes, being as a useful method of preventing the formation or progression of many cardiovascular (CV) diseases, is widely considered valuable [1]. Specific cardiovascular screening is more effective for the detection of pathologic CV states and the prevention of sudden cardiac death. However, CV evaluation is rather expensive, and is mainly available for athletes with presumed pathology. Only a small percentage of asymptomatic athletes have an opportunity to undergo detailed CV screening.

Regarding the referees, the situation may be much worse. In Hungary, regular preparticipation screening and authorization is also obligatory for them in all levels. Although they have similar physical and mental load as athletes during matches; in general, their physical health is much less in focus. Many cases of sudden death on the field affecting referees are well-known from the media. Nevertheless, only few literature data are available referring to the prevalence of CV risk factors and pathologies in referees.

Furthermore, the physical capacity of referees can basically influence decision making in match situations, therefore more attention should be given to improve it [2]. Some information are available about the physical fitness field measurements of referees, especially in outdoor sports, mainly in football. These examinations are part of the regular assessment of their ability to participate as referees during the season. Many options are available to measure their movements, speed and energy expenditure during the games, similarly to athletes, however cardiovascular evaluation is missing [3,4]. In-hospital cardiopulmonary exercise testing is a suitable method of cardiovascular screening combined with standardized physical fitness measurement. Although this examination is highly informative and reproducible, it is not often used in the screening and selection of referees because of its technical difficulties and high expenses. Currently we do not have comprehensive data about the cardiopulmonary function among referees in different kind of sports.

Handball as a mixed sport requires adequate endurance, dynamic performance, and concentration from the athletes and from their referees as well. The men’s and women’s Hungarian National Handball Leagues are among the strongest national leagues in the world, as their top club teams are usually in leading positions in the EHF Champions Leagues.

Within a unique cooperation between the Semmelweis University Heart and Vascular Center (Hungary) and the Hungarian Handball Federation, we had the opportunity to examine the prevalence of cardiac risk factors and diseases among Hungarian elite handball referees who underwent an extended cardiac screening. In our cross-sectional study, we aimed to get a detailed picture about the cardiovascular status of a special, however uncared group of significant contributors of sport events.

## Material and methods

Due to the above-mentioned agreement between the Semmelweis University Heart and Vascular Center and the Hungarian Handball Federation, we had the opportunity to carry out detailed screening of the top one hundred asymptomatic elite handball referees. The selection of the referees was performed by the Referee Subcommittee of the Hungarian Handball Federation, all the referees from the first division were invited, while from the second division those who were the most promising to be promoted in the next years. All the invitations were accepted by the referees. Prior to the study, all participants gave written informed consent to the examinations, and the Medical Research Council of Hungary approved (13687-1/2011-EKU) this study according to the Ethical Guidelines of the Helsinki Declaration. All measurements were performed at least 12 hours after the last training session or refereeing. Subjects with symptoms, or who suspended regular physical activity in the last 6 months were not involved. Personal and family history was taken by a detailed questionnaire and by a personal interview conducted by a cardiologist. Following physical examination and blood pressure measurement, 12-lead ECG was recorded in a resting, lying position and analysed according to the current guidelines (CardioSoft PC, GE Healthcare, Finland) [5]. The evaluation of blood pressure values was carried out in concordance with the current European guideline: resting values over 139 mmHg systolic and/or 89 mmHg diastolic blood pressure were considered hypertensive [6]. The fasting laboratory examination contained complete blood cell count, ions, detailed lipid analysis, liver and kidney panel, iron profile, creatin kinase (CK), lactate dehydrogenase, glucose, haemoglobin A1C, and thyroid panel. All the laboratory examinations were carried out in the same laboratory with the same equipments. Lipid cut-off values were determined as per to the recent ESC/EAS guidelines [7]. The cut-off values for slightly elevated CK were between 190.0 U/l and 500.0 U/l, whereas over 500.0 U/l for markedly elevated CK. Body-composition analyses were carried out by bioelectrical impedance measurements (Bodystat 1500 MDD, Bodystat Ltd, UK; InBody 770, InBody Co. Ltd, South Korea). Routine echocardiography was performed according to the current guidelines (Vivid E95, GE Vingmed Ultrasound, Horten, Norway) [8]. Cardiopulmonary exercise testing was implemented on a treadmill ergometer (T-2100, GE Healthcare, Finland) using an incremental protocol starting with a 1-minute flat walk of 6 km/h, followed by continuous 10 km/h uphill running with an increasing slope of 1.0% every minute until exhaustion. After stopping running, measurements were continued during a 1-minute 4 km/h walk and a further 4-minute rest. Gas analysis was carried out using an automated cardiopulmonary exercise system (Respiratory Ergostik, Geratherm, Germany). When needed, Holter ECG (Cardiospy, Labtech Ltd., Hungary), ambulatory blood pressure monitorization (Card(X)plore, Meditech, Hungary), cardiac magnetic resonance imaging (CMR; Achieva, Philips Medical Systems, The Netherlands) or cardiac computer tomography (CCT; Brilliance iCT256, Philips Medical Systems, The Netherlands) were also performed. The results were analysed by a cardiology expert according to the available guidelines [5–8].

Statistical analysis was performed using a dedicated software (Microsoft Excel, Microsoft Corporation, US), data are presented as mean ± SD, comprehensions were carried out by Fischer exact test, significance was established when p<0.05.

## Results

### Study population

The studied 100 elite handball referees (age: 29.6 ± 7.9 years, age range: 18–46 years, male: 64.0%) trained an average of 4.3 ± 2.0 hours per week (Table 1). In all, 51.0% of them were referees in the first division, and 49% were in the second division of the Hungarian National Handball League. 16% of them were also official referees in either the International or the European Handball Federations. Previously, 39.0% had played handball as athletes in the first or second divisions.

**Table 1:** Main characteristics of the studied referees.

Variable	Mean ± SD	Min—Max
Age (years)	29.6 ± 7.9	18.0–46.2
Training (hours/week)	4.3 ± 2.0	1.0–11.5
Height (cm)	178.0 ± 8.1	162.0–196.0
Body mass (kg)	78.0 ± 13.3	48.0–116.0
BMI (kg/m^2^)	24.5 ± 2.7	18.0–32.8
Body fat (%)	18.7 ± 6.6	4.6–33.2

Abbreviations: BMI, body mass index.

### Personal and family history

None of the examined referees had any CV symptoms. In terms of the CV risk factors or diseases, 24% had positive personal or family history. Sudden cardiac death due to acute myocardial infarction of their parents or grandparents at younger ages was found in the family history in two cases. In one case the personal history was positive due to syncope. At the time of the study, 21.0% of them were smoking (male: 15.0%). Surprisingly, those who played handball previously at higher levels had a higher tendency for smoking compared to the non-elite players (25.6 vs. 18.0%, p = 0.45). They had no prior established cardiovascular diseases, except for treated arterial hypertension in 4.0% and ablated AV re-entry tachycardia in 1.0%.

### Physical examination

No significant physical abnormalities were found during routine examinations. Mean height, body mass, body mass index (BMI) and body fat percentage are shown on Table 1. Without increased BMI, 5.0% of the referees proved to have an isolated increase in body fat percentage. Due to fat-free mass increase, 31.0% had higher BMI values with having their body fat values in the normal range. Considering both higher body fat and BMI increase, 10.0% were overweight (male: 9.0%), whereas obesity was diagnosed in 3.0%, all of them were male. Obesity and overweight had a tendency to be more frequent between those referees who did not play previously in elite levels compared to those who played (16.4 vs 7.7%, p = 0.24).

Resting mean systolic blood pressure was measured as 133.5 ± 16.2 mmHg, while resting mean diastolic blood pressure was 82.4 ± 10.5 mmHg. Isolated elevation of systolic blood pressure was found in 17.0% of the referees, while 9.0% had isolated diastolic blood pressure elevation. Both systolic and diastolic resting blood pressures were higher in 12.0%. Elevated values of resting blood pressure were measured more often at the former non-elite handball player group compared to the former elite players (49.2 vs 20.5%, p = 0.006).

### Resting ECG

Mean resting heart rate was 78.8 ± 11.8 b.p.m. On the resting 12-lead ECG recordings, isolated QRS voltage criteria for left ventricular hypertrophy were found in 2.0%, incomplete right bundle branch block in 47.0%, sinus bradycardia in 10.0%, sinus arrhythmia in 6.0%, first degree atrioventricular block in 2.0% as normal, sports-related ECG changes. Out of the 55 referees who had at least one sport-related physiological ECG changes, 37 were male. Right axis deviation appeared in 2.0%, left axis deviation in 14.0%. These grey zone ECG changes were isolated in all of the cases, requiring no further evaluation according to the current guidelines. Pathological inferior ST-depression was found in 1.0%, and pathological T-wave inversion or biphasic T-waves were recorded in 6.0% of the cases. Sinus tachycardia was detected in 2.0% (heart rate: 102 b.p.m. and 130 b.p.m.) on the resting ECG, out of which bigeminous monomorphic ventricular extrasystoles were also recorded in one case. No other grey zone or pathological ECG abnormalities were detected on the 12-lead ECG. In total, 10 referees had one or two pathological ECG changes, and four of them were male.

### Laboratory examinations

Due to physical activity, mean level of serum creatine kinase slightly elevated. Individually, slightly elevated values were measured in 26.0%, and markedly elevated values, in 4.0%. Dyslipidaemia requiring lifestyle changes was found in 38 patients, 33 out of them were male, lipid-lowering medication was indicated in 3 cases. The measured values of the laboratory blood examinations are represented in the Table 2.

**Table 2:** Results of the blood examinations.

	Mean ± SD	Reference value
Creatine kinase (U/l)	230.2 ± 399.1	3.0–190.0
Total cholesterol (mmol/l)	4.6 ± 0.8	0.0–5.2
LDL cholesterol (mmol/l)	2.9 ± 0.8	0.1–3.3
Triglycerides (mmol/l)	1.1 ± 0.8	0.0–2.2
HDL cholesterol (mmol/l)	1.6 ± 0.4	0.9–1.4
Free iron (mmol/l)	18.5 ± 7.4	12.5–32.2
Total iron-binding capacity (umol/l)	70.5 ± 13.0	45.0–81.0
Transferrin (g/l)	2.9 ± 0.5	2.0–3.6
Transferrin saturation (%)	23.0 ± 11.8	16.0–45.0
Ferritin (ug/l)	112.0 ± 96.8	20.0–250.0
Red blood cell (10^12^/l)	4.9 ± 0.4	4.3–5.9
Haemoglobin (g/l)	145.8 ± 15.5	130.0–180.0

Abbreviations: SD, standard deviation.

Decreased free iron levels were found in 20.0% of the referees, while total iron-binding capacity increased in 16.0%, transferrin increased in 5.0%, transferrin saturation decreased in 16.0% and ferritin level decreased in 6.0% of the cases. Lower red blood cell count was found in nine female referees, while lower haemoglobin level in nine females as well. All these cases were attributed to iron deficiency. No other significant laboratory test deviations were found.

### Systematic coronary risk estimation

Most of the referees, 96.0% had a Systematic Coronary Risk Estimation (SCORE) point <1, while four referees had a SCORE point between ≥1 and < 5.

### Echocardiography

Average left ventricular ejection fraction was measured as 59.9 ± 3.3%. Grey zone posterior end diastolic wall thickness was found in 2.0% (average: 8.6 ± 1.4 mm), and grey zone interventricular septal wall thickness in 3.0% (average: 9.5 ± 1.5 mm), all these changes were found in males. In terms of the pathological changes, first grade mitral valve insufficiently was seen in 6.0%, and mitral prolapse syndrome in 3.0%. Interatrial septal aneurysm was detected without shunt flow in one patient and increased trabecularization was seen in another case. Dilated aortic root was detected in 2.0% of the cases, one out of these patients also had bicuspid aortic valve. No cases of functional deviation were found regarding the right ventricles, but one athlete had increased right ventricular dimensions. The pathological echocardiographic changes affected 6 male and 6 female patients.

### Cardiopulmonary exercise testing

Applying our sport-specific protocol, the average running time was 9.3 ± 2.8 min. The measured average maximal relative aerobic capacity was 44.9 ± 6.5 ml/kg/min. The most common pathological changes were hypertensive exercise or restitution blood pressure values in 10.0%, multiple premature ventricular contractions (PVC) during exercise or during recovery in 8.0%, and significant ST-T changes in 8.0% of the referees. Heart rate-dependent left bundle branch block was detected in one patient, decreased maximal aerobic capacity was found in another patient.

### Additional examinations

**Fig 1:**
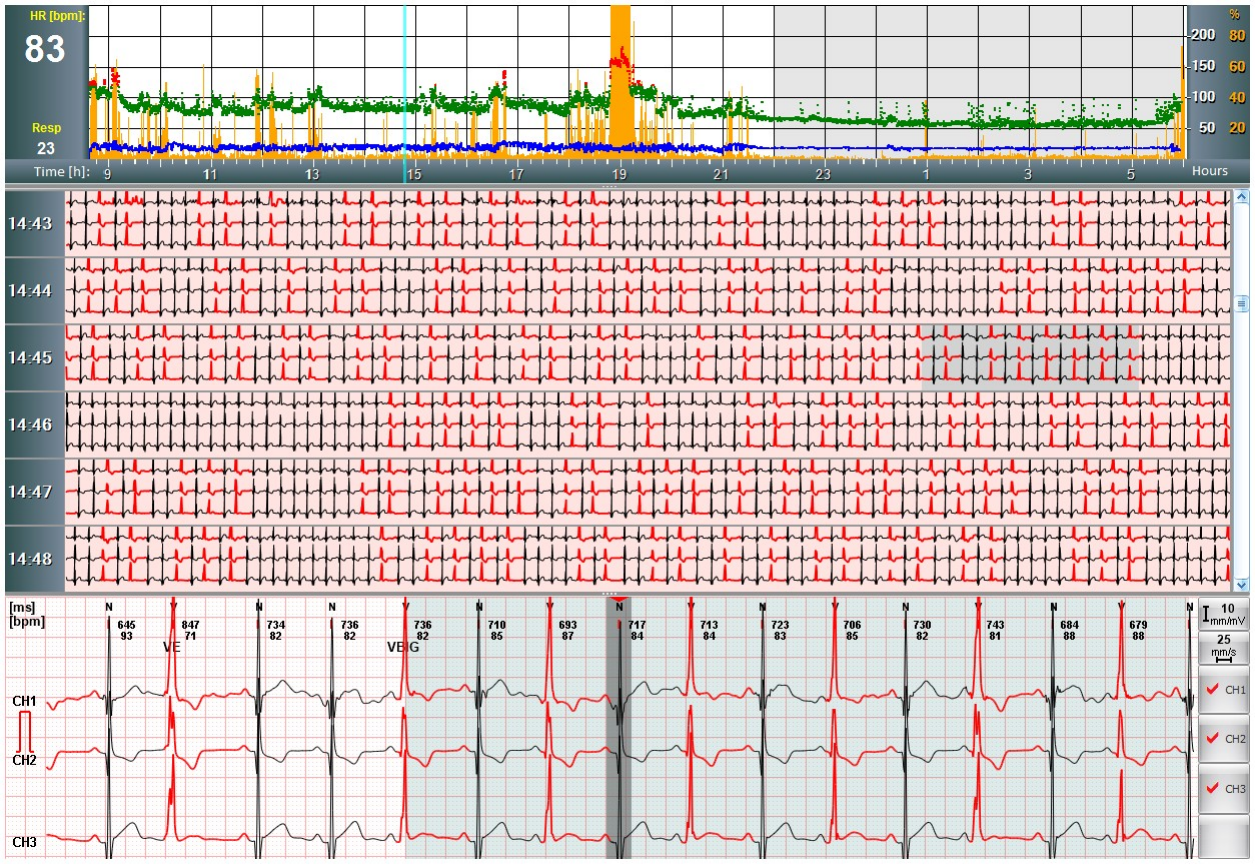
Premature ventricular contractions of a male referee on Holter ECG. Holter ECG recording of a 22-year-old asymptomatic male handball referee performed due to exercise induced single premature ventricular contractions. Heart rate (normal: Green, tachycardia: Red), respiration rate (blue) and movements (orange) are shown against time at the top of the figure. The high number of premature ventricular contractions (red) is presented between normal beats (black) during a resting daytime period on the middle part of the picture. A representative section of the recording shows ventricular bigeminy on the Holter ECG channels 1, 2 and 3 (modified V5, V1 and III) at the bottom. The examination revealed 9.4% single monomorphic premature ventricular contractions, numerous episodes of ventricular bigeminy and ventricular triplets. Echocardiography and CMR examinations proved to be normal. Beta-blocker therapy was initialized, and regular cardiology follow-up was indicated. Abbreviations: CMR, cardiac magnetic resonance; HR, heart rate; bpm, beats per minute; Resp, respiration; h, hour; N, normal beat; V, ventricular beat; CH, channel. Signal amplitude: 10 mm/mV, paper speed: 25 mm/sec.

Holter ECG recordings were carried out in seven cases; a significant number of PVCs was found in two cases (PVC: 9.4% and PVC: 1.6%), one out of these patients also had numerous bigeminous premature ventricular contraction episodes and ventricular triplets (Fig 1). In total, 10 ambulatory blood pressure monitoring (ABPM) examinations were indicated; hypertension was diagnosed in two cases, undertreated hypertension in one case, slightly elevated blood pressure values in two cases, all of these patients were male. Normal blood pressure values were measured with ABPM in three cases, while two patients did not undergo the examinations for their personal decisions.

**Fig 2:**
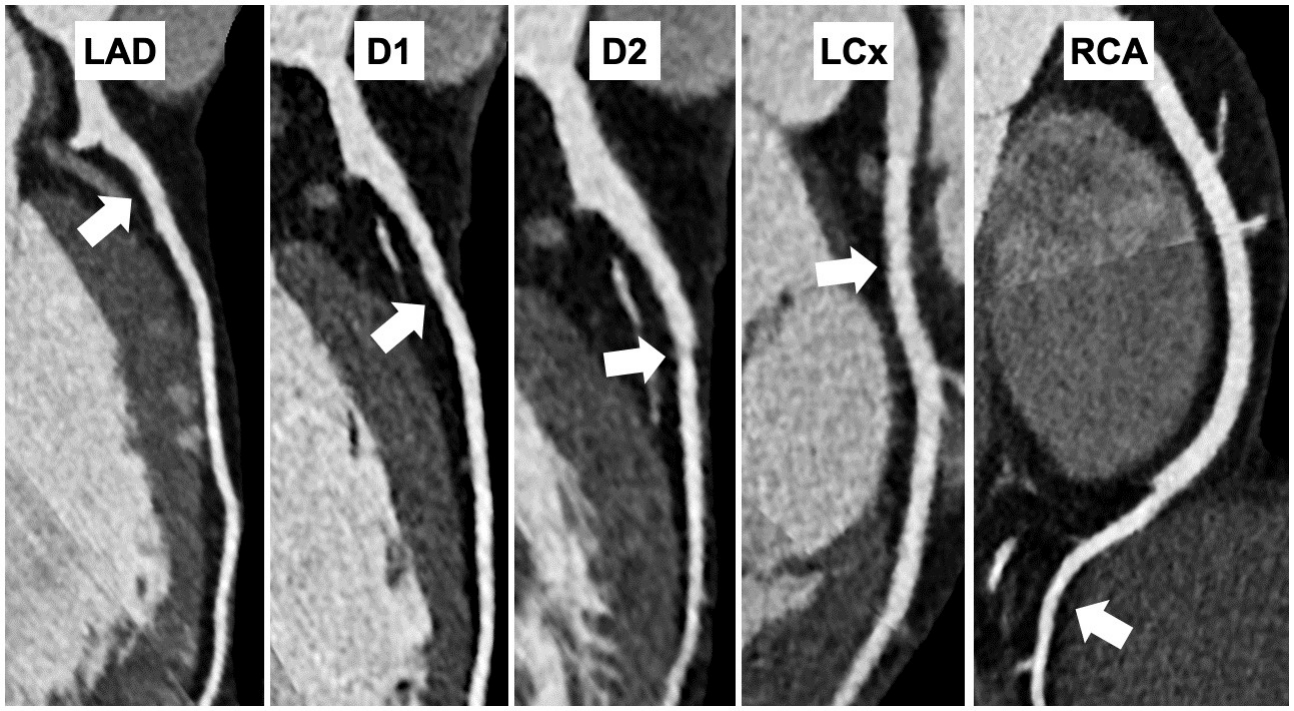
CCT of a male referee with non-significant coronary artery atherosclerosis. The CCT pictures of a 45-year-old asymptomatic male handball referee with no cardiovascular risk factors except for slightly elevated LDL cholesterol level. The examination was performed due to significant lateral ST-T changes recorded during exercise stress testing and revealed non-significant low-density non-calcified atherosclerotic plaques in all main coronary arteries (arrows). Statin therapy was initiated, and regular cardiology follow-up was indicated. Abbreviations: CCT, cardiac computer tomography; LAD, left anterior descending artery; D1, first diagonal artery; D2, second diagonal artery; LCx, left circumflex artery; RCA, right coronary artery.

So far, a total of 27 referees underwent CMR, due to offered screening in 12 cases and for diagnostic indications in 15 cases. Diagnostic CMR examinations were carried out because of personal history of syncope in one case, minor echocardiography changes in three cases (left ventricular hypertrophy: 1, interatrial septal aneurism: 1, enlarged right ventricular dimensions: 1), and resting or exercise ECG changes in 11 cases (frequent ventricular premature beats: 4, ST-T changes: 6, heart rate dependent left bundle branch block: 1). Hypertrabecularization was seen in two cases, borderline left and right ventricular functions in three cases—one out of them also had wall motion abnormality. One case of aortic dilatation and bicuspid aortic valve, previously found with echocardiography, was confirmed by CMR. In case of one referee, circular pericardial contrast enhancement referring to previous pericardial inflammation was detected. A CCT was carried out in four cases due to wall motion abnormality in one case, and stress ECG ST-T abnormalities and PVCs in three cases. Non-significant coronary artery atherosclerosis was diagnosed in one case (Fig 2) and LAD bridge was found in another. Two more patients with CCT indications have not yet undergone the examinations because of their personal decisions.

### Interventions

Lifestyle changes were advised to 58.0% of the referees, including quitting smoking, losing weight, and introducing dietary changes (Table 3). New antihypertensive drug therapy was offered in 2 cases, antihypertensive drug therapy modification in 1 case, regular blood pressure monitorization in 12 cases. Lipid-lowering therapy was started in three male patients because of elevated lipid levels in all cases and also a non-significant coronary artery disease in one case. Beta-blocker therapy was initialized in one patient for the treatment of frequent PVCs. Oral iron supplementary therapies and control blood testing were suggested in 22.0% (male: 9.0%). Regular cardiology control examinations were recommended to 29 patients. Recommendation of lifestyle changes was more often in male referees, iron supplementation was suggested in more female referees. No difference was found between former elite and former non-elite referees regarding the number of interventions indicated.

**Table 3:** Number of interventions indicated by the extended cardiology screening of handball referees.

Interventions	Comprehension of sexes (N)			Comprehension of previous level as a player (N)		
	Male (N = 64)	Female (N = 36)	p	Former elite (N = 39)	Former non-elite (N = 61)	p
Lifestyle changes	42	16	0.06	23	35	1.00
• Stopping smoking	15	6	0.61	10	11	0.45
• Losing weight	12	1	**0.03**	3	10	0.24
• Dietary advice	37	11	**0.01**	18	30	0.84
Oral iron supplementation	9	13	**0.02**	9	13	1.00
Antihypertensive drug therapy (new or modified)	3	0	0.55	1	2	1.00
Lipid lowering drug therapy	3	0	0.55	1	2	1.00
Rhythm control drug therapy	1	0	1.00	1	0	0.39
Cardiology follow-up	20	9	0.65	11	18	1.00

Abbreviations: SD, standard deviation. Comprehension by Fisher exact test; significance: p<0.05.

## Discussion

According to the available data, we report the first results of a detailed cardiology screening focusing on the prevalence of CV risk factors and diseases among handball referees. In general, we only have limited resources in the literature about the prevalence of CV risk factors and CV diseases in referees. However, we have some available data about referees of other kinds of sports, especially football.

Comparing our results with a slightly older age group of the general population of Hungary based on the *Budakalász study*, we found lower BMI, similar systolic and slightly higher diastolic blood pressure and lower rates of dyslipidaemia in handball referees. In our referee population the rate of smokers was slightly lower comparing to the same age group in the general population [9]. In a recently published article data of a 30–40 year-old Hungarian sedentary population are available [10]. They found that 29.5% of the examined population was smoking, 11.5% of them was treated for high blood pressure and none of them was receiving lipid lowering therapy. In our referee population the percentages of smoking and antihypertensive treatment were lower, while higher percentage of lipid-lowering therapy was indicated.

In terms of the CV risk factors or diseases, a high percentage, almost one quarter of Hungarian handball referees had positive personal history, mostly because of smoking. Smoking rate was as high as 21.0% among Hungarian handball referees. By the results of O’Riordan et al., smoking rate was higher, 35.0% among male Irish Gaelic athletic sports referees, while it was lower, 13.0% in professional Ukrainian football referees, according to Tereshchenko et al [11,12]. These results raise our attention to the surprisingly high percentage of tobacco use among referees who are thought to strive to maintain their health. The basic characteristics and the most important findings of our study and the mentioned previous referee examinations are shown on Table 4.

**Table 4:** Comprehension of different referee studies.

Study	Population size (male, %)	Age (mean ± SD)	Sport of refereeing	Smoking (%)	Overweight or obesity (%)	Hypertension (%)	Dyslipidaemia (%)	Decreased iron storage (%)	Pathological ECG (%)	CPET changes (%)	Echo changes (%)
Kiss et al.	100 (61)	29.6 ± 7.9	Handball	21.0	13.0	6.0	41.0	22.0	10.0	19.0	12.0
O’Riordan et al.	183 (100)	45	Gaelic athletic sports	35	85.8	6	43.2	-	-	-	-
Tereshchenko et al.	174	30.7 ± 0.4	Football	13	10.3	10.2	-	-	-	-	-
Bizzini et al.	90 (100)	39.1 ± 3.9	Football	-	-	6.7	2.2	2.2	4.4	5.6	0
Loureiro da Silva et al.	16 (87.5)	34.2 ± 4.1	Football	-	0	-	56.3	-	-	-	-
Keller et al.	51 (0)	33.2 ± 3.8	Football	-	-	3.9	-	-	0	-	7.8

Abbreviations: CPET, cardiopulmonary exercise testing; ECG: Electrocardiogram.

Four of our patients had treated hypertension, additionally, our screening revealed two other cases of hypertension requiring medical treatment. As compared to the sum of 6.0% treated hypertension in our handball referee population, Bizzini et al. found a treated hypertension rate of 1.1%, and an exercise hypertension rate of 5.6% among male FIFA football referees [13]. Moreover, Tereshchenko et al. discovered arterial hypertension in 10.2% among professional Ukrainian football referees [12].

Another important result of our study is finding 10.0% of elite handball referees overweight and 3.0% of them obese. Although some of the available studies examining referees present average BMI and body fat percent data, these do not define the percentage of overweight and obese referees. O’Riordan et al. report a remarkable 60.1% of overweight and a 25.7% of obese patients between Irish Gaelic athletic sports referees. However, these data were determined simply based on BMI results without using body composition analysis [11]. Moreover, Loureiro da Silva et al. found 44% of Brazilian football referees overweight based on BMI and adipometer measurements [14]. Our particular results are more favourable; however, they still highlight the need for optimizing the body composition of every seventh elite handball referee. Furthermore, in case of athletes, BMI measurement is not sufficient for the assessment of excess body weight.

Despite the fact, that regular training could help lowering serum cholesterol levels, we revealed dyslipidaemia in more than 40% of our elite handball referees. This percentage is very high compared to the results of male FIFA football referees (2.2% elevated serum lipid levels), although we do not know the exact cut-off values [13]. Otherwise, a similar 37.5% of Brazilian football referees presented dyslipidaemia, and they had a higher average of total lipid levels compared to our population with nearly the same HDL cholesterol levels [14]. These differences could suggest that the Brazilian football referees had higher average LDL cholesterol and triglycerides, which could be related to the above-mentioned higher prevalence of overweight in this group.

Surprisingly, decreased iron storage levels were found in one fifth of the referees and nearly one tenth of the referees also had iron deficiency anaemia requiring oral iron supplementation. On the contrary, Bizzini et al. found just 2.2% of mild anaemia in their male football referee population [13]. The difference between the two studies could come from the different gender composition, since in our study, most of the referees who had iron deficiency were women. Since in our study the anaemias were slight and the referees did not report any symptoms, which could be caused by these changes, dietary deviation was assumed, oral iron supplementation was indicated, and control blood tests were recommended after a 3-month-long treatment.

Analysing the resting 12-lead ECG, we found physiological, sport-related changes in a large proportion of the referees, referring to regular training. Isolated grey zone changes requiring no further evaluations occurred in one-sixth of the referees. In terms of pathological ECG changes, T-wave inversion or biphasic T-waves were the most frequent abnormalities. These results correlate with the findings in both female and male FIFA referees: physiological sport-related changes were common in both studies [13,15]. In terms of pathological changes, they did not find any significant changes in the female football referee population, while T-wave inversion was recorded in 4.4% among the male referees. Out of our nine patients having pathological resting ECG abnormalities, one was diagnosed with significant PVC number, one with pericardial contrast enhancement, and one with hypertension, while six patients (4 having ST-T abnormalities, 2 having resting sinus tachycardia) had no CV pathologies.

During the cardiopulmonary exercise testing, we measured a good average maximal aerobic capacity among the Hungarian handball referees. Although Fernandes da Silva et al. detected a nearly 8% better aerobic capacity among elite handball referees in Brazil, their results were estimated by an equation, while our results were measured directly from breath-by-breath measurements [16]. Furthermore, Loureiro da Silva et al. using breath-by-breath analysis measured a maximal oxygen uptake in football referees similar to our results [14]. The exercise testing reported hypertensive blood pressures in one-tenth of the examined referees. The most common ECG abnormalities were ST-T changes and multiple PVCs during exercise or restitution, both occurred in 8.0%. Heart rate-dependent left bundle branch block was observed in one patient. Bizzini et al. found exercise-induced ST depression in their football referee population in 5.6%, and no other exercise ECG changes were detected in their study [13].

Echocardiographic examinations showed an average of 59.9 ± 3.3% left ventricular ejection fraction with an average posterior end-diastolic wall thickness of 8.6 ± 1.4 mm, and an intraventricular septal wall thickness of 9.5 ± 1.5 mm. In comparison, Caballero et al. described higher ejection fraction and thicker posterior end-diastolic wall and septal wall parameters (68.7 ± 8.9%, 9.5 ± 1.6 mm, 9.77 ± 1.5 mm, respectively) in Spanish football referees [17]. Keller et al. measured higher left ventricular ejection fraction with thinner posterior and septal end-diastolic wall in FIFA referees (67.6 ± 4.2%, 8.3 ± 1.0 mm, 7.9 ± 1.0 mm, respectively), however they were all women [15]. In our study, grey zone wall thickness was found in 2.0% at the posterior wall and in 3.0% at the septal wall, while in the Spanish study, they did not measure more than 12 mm at either wall [17]. In our population, the most common pathological echocardiographic changes were first-grade mitral valve regurgitation in 6.0% and mitral prolapse syndrome in 3.0%. Other pathologies were rare, including single cases of interatrial septal aneurysm and hypertrabecularization, enlarged right ventricle dimensions in one referee, dilatation of the aortic root in two patients, with bicuspid aortic valve in one out of these. Among female FIFA referees, the most common echocardiographic changes were mitral valve regurgitation in 17.6%, other changes were rare, such as aortic regurgitation in 2.0% and ventricular septal defect in 3.9% [15]. In male FIFA referees, the rate of valvulopathies was lower, mitral regurgitation was detected in 2.2%, mitral prolapse syndrome in 1.1%, while both dilatation of the aortic root and left ventricular hypertrabeculation were detected in 2.2% in this population [13].

Additional examinations were not an obligatory part of our study and were principally carried out for further evaluation of potential diseases, except for the CMR, which were also offered to the referees without any suspicious changes for scientific purposes. By Holter ECG recording, significant ventricular arrhythmias were found in two cases (Fig 1), while new hypertension was diagnosed in two patients and undertreated hypertension in one patient by ambulatory blood pressure measurement examinations. The CMR examinations revealed hypertrabecularization in two cases, borderline left and right ventricular functions in three cases, wall motion abnormality in one case, pericardial contrast enhancement in one case. Three out of the above-mentioned cases underwent CMR examination for screening reasons without any pathological signs detected during the previous screening. The CCT detected left anterior descending coronary bridge in one case. Non-significant coronary artery atherosclerosis was diagnosed by CCT after detecting exercise ECG changes in a 45-year-old asymptomatic male patient with no cardiovascular risk factors except for a slightly elevated LDL cholesterol level (Fig 2). Coronary artery disease can lead to acute myocardial infarction and sudden death also in cases of non-significant lesions. Important trigger factors of these acute events include strenuous exercise or psychological stress during sports events, therefore it is of great significance to detect referees having coronary artery disease in such an early phase. In summary, 46.5% of the detected CV risk factors or diseases had no signs or symptoms and were not diagnosed during routine screening, they were only revealed by the extended screening (Table 5). Out of this 46.5%, more than half of the cases resulted from dyslipidaemia. Moreover, laboratory screening surprisingly frequently verified iron deficiency.

**Table 5:** Number of CV risk factors and pathologies of the handball referees revealed by routine sports screening and extended cardiovascular screening.

CV risk factors and pathologies	Signs revealed by routine sports screening (N)	Signs only revealed by extended cardiovascular screening (N)
Positive family history	2	
Smoking	21	
Previously detected hypertension	4	
Previous AVRT ablation	1	
Overweight	10	
Obesity	3	
Hypertension I-III. stage	38	1
Dyslipidaemia		41
Iron deficiency		21
Significant PVC number		2
Heart rate dependent LBBB		1
Grey zone ventricular wall thickness		3
Mitral valve prolapse		6
Mitral valve insufficiency		1
Interatrial septal aneurysm		1
LV hypertrabecularization		2
Borderline LV/RV function		3
Aortic root dilatation		2
Bicuspid aortic valve		1
Pericardial contrast enhancement		1
LAD bridge		1
Coronary artery disease		1

Abbreviations: CV, cardiovascular; AVRT, AV re-entry tachycardia; PVC, premature ventricular contraction; LBBB, left bundle-branch block; LV, left ventricle; RV, right ventricle; LAD, left anterior descending artery.

These results confirm the necessity of extended cardiology screening in this population. With the optimal screening, treatment and follow-up of the above-mentioned pathologies, their progression could be controlled, and severe CV events could be prevented, while their negative effects on health and performance could be reversed.

## Supporting information

S1 Dataset Minimal dataset of the examined referees. (XLSX)
